# RSPO3 induced by *Helicobacter pylori* extracts promotes gastric cancer stem cell properties through the GNG7/β‐catenin signaling pathway

**DOI:** 10.1002/cam4.7092

**Published:** 2024-04-05

**Authors:** Xiwu Rao, Zhipeng Zhang, Yunzhou Pu, Gang Han, Hangjun Gong, Hao Hu, Qing Ji, Ningning Liu

**Affiliations:** ^1^ Department of Oncology The First Affiliated Hospital of Guangzhou University of Chinese Medicine, Guangzhou University of Chinese Medicine, Postdoctoral Research Station of Guangzhou University of Chinese Medicine Guangzhou China; ^2^ Department of Oncology Shuguang Hospital, Shanghai University of Traditional Chinese Medicine Shanghai China; ^3^ Department of Gastroenterology Shuguang Hospital, Shanghai University of Traditional Chinese Medicine Shanghai China

**Keywords:** cancer stem cell, G protein subunit gamma 7, gastric cancer, *Helicobacter pylori*, R‐spondin 3

## Abstract

**Background:**

*Helicobacter pylori* (*H. pylori*) accounts for the majority of gastric cancer (GC) cases globally. The present study found that *H. pylori* promoted GC stem cell (CSC)‐like properties, therefore, the regulatory mechanism of how *H. pylori* promotes GC stemness was explored.

**Methods:**

Spheroid‐formation experiments were performed to explore the self‐renewal capacity of GC cells. The expression of R‐spondin 3 (RSPO3), Nanog homeobox, organic cation/carnitine transporter‐4 (OCT‐4), SRY‐box transcription factor 2 (SOX‐2), CD44, Akt, glycogen synthase kinase‐3β (GSK‐3β), p‐Akt, p‐GSK‐3β, β‐catenin, and G protein subunit gamma 7 (GNG7) were detected by RT‐qPCR, western blotting, immunohistochemistry (IHC), and immunofluorescence. Co‐immunoprecipitation (CoIP) and liquid chromatography coupled with tandem mass spectrometry (LC–MS/MS) were performed to identify proteins interacting with RSPO3. Lentivirus‐based RNA interference constructed short hairpin (sh)‐RSPO3 GC cells. Small interfering RNA transfection was performed to inhibit GNG7. The in vivo mechanism was verified using a tumor peritoneal seeding model in nude mice.

**Results:**

*H. pylori* extracts promoted a CSC‐like phenotype in GC cells and elevated the expression of RSPO3. RSPO3 knockdown significantly reduced the CSC‐like properties induced by *H. pylori*. Previous studies have demonstrated that RSPO3 potentiates the Wnt/β‐catenin signaling pathway, but the inhibitor of Wnt cannot diminish the RSPO3‐induced activation of β‐catenin. CoIP and LC–MS/MS revealed that GNG7 is one of the transmembrane proteins interacting with RSPO3, and it was confirmed that RSPO3 directly interacted with GNG7. Recombinant RSPO3 protein increased the phosphorylation level of Akt and GSK‐3β, and the expression of β‐catenin in GC cells, but this regulatory effect of RSPO3 could be blocked by GNG7 knockdown. Of note, GNG7 suppression could diminish the promoting effect of RSPO3 to CSC‐like properties. In addition, RSPO3 suppression inhibited MKN45 tumor peritoneal seeding in vivo. IHC staining also showed that RSPO3, CD44, OCT‐4, and SOX‐2 were elevated in *H. pylori* GC tissues.

**Conclusion:**

RSPO3 enhanced the stemness of *H. pylori* extracts‐infected GC cells through the GNG7/β‐catenin signaling pathway.

## 1 INTRODUCTION

Carcinogenesis is potentially accompanied by chronic infections. *Helicobacter pylori* (*H. pylori*) can be found in at least half of the world's human population, and accounted for ~810,000 cases of gastric cancer (GC) worldwide in 2018.^1^ *H. pylori* has therefore been classified as the most important carcinogen leading to GC.^2^, ^3^ Reports have demonstrated that *H. pylori* facilitates GC invasion^4^ and stemness,^5^, ^6^, ^7^ and it may be associated with the induction of slender and sharp cell morphology called the “hummingbird” phenotype.^8^ This phenotype has been reported to be accompanied by epithelial–mesenchymal transition (EMT),^7^ during which confers GC stem cell (CSC)‐like properties on otherwise epithelial carcinoma cells.^9^ CSCs correspond to a subpopulation of cells with a self‐renewal capacity responsible for initiating tumor growth and evolution.^10^ CSCs increase sphere formation in a suspension culture, and enhance stemness to seed tumors in mice.^11^ Certain specific cell‐surface markers, such as CD44 and SRY‐box transcription factor 2 (SOX‐2),^10^ Nanog homeobox (Nanog),^12^ and organic cation/carnitine transporter‐4 (OCT‐4)^13^ can evaluate the degree of stemness.

Wnt signaling regulates cancer stemness by a complex network of β‐catenin‐binding factors.^14^ In an inactive Wnt signaling state, the phosphorylated β‐catenin degraded by the destruction complex, which containsadenomatous polyposis coli, axis inhibition protein (Axin), glycogen synthase kinase‐3β (GSK‐3β), casein kinase (CK1α). But when Wnt ligands bind to Frizzled receptors and LDL‐receptor‐related proteins (LRP), LRP receptors are then phosphorylated by GSK‐3β and CK1α, and disheveled (Dvl) proteins are recruited to the membrane.^15^ The Dvl polymers inhibit the destruction complex, release the β‐catenin, and then translocates into the nucleus, which recruits histone‐modifying co‐activators. This transcriptional process interferes with multiple cellular processes.^14^, ^16^

R‐spondins (RSPOs) are secreted proteins, it bind to the leucinerich repeat containing G‐protein coupled receptors (LGRs) to potentiate Wnt signaling.^17^, ^18^, ^19^ R‐spondin 3 (RSPO3) promotes stem‐cell function in colorectal cancer^20^ and acute myeloid leukemia,^21^ and its overexpression may lead to cancer development and poor survival.^22^, ^23^ Of note, RSPO3 orchestrates gastric epithelial stem cells,^24^ and *H. pylori* stimulates the secretion of RSPO3 by myofibroblasts, which then increases AXIN2^+^LGR5^−^ stem cell proliferation.^25^ Coincidentally, it was found that *H. pylori* stimulated RSPO3 in GC cells, and promoted CSC properties; thus, the regulatory mechanism through which RSPO3 upregulates GC stemness, and whether this is associated with Wnt signaling were explored.

## 2 MATERIALS AND METHODS

### 2.1 Preparation of *H. pylori* extracts

Renji Hospital (Shanghai, China) provided the *H. pylori* strain NCTC11637 (containing cacA and cagA gene). *H. pylori* was cultured on Columbia agar plates (Oxoid, Basingstoke Hampshire, UK) containing 5% sheep blood at 37°C. *H. pylori* lysate was prepared as previously described.^2^, ^26^ *H. pylori* was suspended in phosphate buffered saline (PBS) and pulse‐sonicated in an ice bath. Centrifuge suspension to remove bacterial debris and filter the collected supernatant through a cellulose acetate filter for sterilization. The lysates were determined the protein concentration by BCA protein assay (cat. no. P0011; Beyotime Institute of Biotechnology, China) and stored at −80°C.

### 2.2 Cell culture

AGS cell line was obtained from the Chinese Academy of Sciences and an MKN45 cell line was obtained from the American Type Culture Collection. Both AGS and MKN45 cells were cultured in RPMI 1640 medium (cat. no. L210KJ, BasalMedia, Shanghai, China) containing 10% fetal bovine serum (cat. no. S660JJ, BasalMedia, Shanghai, China), and 1% streptomycin and penicillin (cat. no. S110JV, BasalMedia, Shanghai, China), at 37°C with 5% CO_2_ and saturated humidity. Cells were treated with *H. pylori* extracts or recombinant human RSPO3 (100 ng/mL, cat. no. KL173Hu011, KALANG, Inc., USA).

### 2.3 Lentivirus‐based RNA interference

Lentiviral vectors expressing RNA interference sequences specifically targeting short hairpin (sh)‐RSPO3 were obtained from the Genomeditech (Shanghai, China). The targeted sequences were as follows: sh‐NC, 5′‐UUCUCCGAACGUGUCACGUdTdT‐3′ sense and 5′‐ACGUGACACGUUCGGAGAAdTdT‐3′ antisense; sh1‐RSPO3, 5′‐GCATCCTAACGTTAGTCAAGG‐3′; sh2‐RSPO3, 5′‐GGATATTATGGAACTCGATAT‐3′; sh3‐RSPO3, 5′‐GGAGTCCATGCACGAAGAAGG‐3′.

### 2.4 Cell transfection

Cell transfection was performed according to the HilyMax kit instructions (cat. no. H357, Dojindo Molecular Technologies, Inc., Japan). G protein subunit gamma 7 (GNG7)‐targeting small interfering RNA GNG7 (si‐GNG7) was provided by Genomeditech (Shanghai, China). Non‐targeting siRNAs were used as negative controls. The si‐GNG7 was introduced into cells at a concentration of 100 nM. The sequences of siRNA were as follows: si‐NC, 5′‐UUCUCCGAACGUGUCACGUdTdT‐3′ sense and 5′‐ACGUGACACGUUCGGAGAAdTdT‐3′ antisense; si‐GNG7‐1, 5′‐GGAACAGCUACGCAUAGAAtt‐3′; si‐GNG7‐2, 5′‐GACAAGAAACCUUGUAUUAtt‐3′; si‐GNG7‐3, 5′‐CAUGAGCUACUGUGAGCAAtt‐3′.

### 2.5 Self‐renewal assay

AGS and MKN45 cells were seeded in 6‐well ultra‐low attachment plates (cat. no. 3471, Corning, Inc., USA) and cultured in RPMI 1640 serum‐free medium, supplemented with 20 ng/mL human recombinant epidermal growth factor and basic fibroblast growth factor (cat. no. 96‐100‐18B‐10, PeproTech, Inc., USA), B27 (dilution, 1:50) supplements (cat. no. 17504044, Invitrogen; Thermo Fisher Scientific, Inc., USA). The cells were cultured in 5% CO_2_ at 37°C for 2 weeks, and the quantity and size of spheroids was counted and measured by microscope. Sphere formation was calculated by counting number of spheres measuring ≥90 μm, expressed as a percentage.

### 2.6 Reverse transcription‐quantitative PCR (RT‐qPCR)

Total RNA was reverse‐transcribed using a PrimeScript RT Reagent Kit (cat. no. P611‐01, Vazyme Biotech Co., Ltd., China) to synthesize cDNA. Quantification of RSPO3, GNG7, CD44, SOX‐2, Nanog, and OCT‐4 expression was normalized to GAPDH expression. RT‐qPCR was performed using the following primers: RSPO3, 5′‐TGGAAGCCAACAACCATACTAT‐3′ sense and 5′‐ACATGTTTTTCCCTTCTTCGTG‐3′ antisense; GNG7, 5′‐CTGGTGG AACAGCTACGCATAGAAG‐3′ sense and 5′‐CCGGGCATGTTGCTCACAGTA G‐3′ antisense; CD44, 5′‐GGGAGTCAAGAAGGTGGAGCAAAC‐3′ sense and 5′‐GCCAAGAGGGATGCCAAGATGATC‐3′ antisense; SOX‐2, 5′‐CAGCATGTCCTACTCGCAGCAG‐3′ sense and 5′‐CTGGAGTGGGAGGAAGAGGTAACC‐3′ antisense; Nanog, 5′‐AGATGCCTCACACGGAGACTGTC‐3′ sense and 5′‐TGGGTTGT TTGCCTTTGGGACTG‐3′ antisense; OCT‐4, 5′‐GAGAACCGAGTGAGAGGCAACC‐3′ sense and 5′‐CATAGTCGCTGCTTGATCGCTTG‐3′ antisense; GAPDH, 5′‐GACAACGAATTTGGCTACAGC‐3′ sense and 5′‐GATGGTACATGACAAGGTGC‐3′ antisense.

### 2.7 Western blotting

Expressions of RSPO3, GNG7, CD44, SOX‐2, Nanog, OCT‐4, and β‐catenin proteins were evaluated using western blotting. Cells were lysed in 1xRIPA buffer (cat. no. P0013B, Beyotime Institute of Biotechnology, China), and lysates were quantified, electrophoresed and transferred onto PVDF membranes, followed by blocking in 10% skim milk for 1.5 h and incubation with an antibody against RSPO3 (cat. no. 17193‐1‐AP, ProteinTech Group, Inc., USA), GNG7 (cat. no. DF9562, Affinity, Inc., USA), CD44 (cat. no. 37259S, Cell Signaling Technology, Inc., USA), SOX‐2 (cat. no. 3579S, Cell Signaling Technology, Inc., USA), Nanog (cat. no. 8822S, Cell Signaling Technology, Inc., USA), OCT‐4 (cat. no. 2750S, Cell Signaling Technology, Inc., USA) and β‐catenin (cat. no. 8480S, Cell Signaling Technology, Inc., USA), for over 12 h at 4°C. Following incubation with the secondary antibody (cat. no. A0208, A0216, Beyotime Institute of Biotechnology, China), protein bands were visualized by ECL detection kit (cat. no. P0018S, Beyotime Institute of Biotechnology, China).

### 2.8 Co‐immunoprecipitation (CoIP)

CoIP was performed according to the CoIP Kit (cat. no. P2055, Beyotime Institute of Biotechnology, China). Cellular lysate was prepared using IP lysis buffer, and 200 μg protein was incubated with 1 μg rabbit IgG and 20 μL fully resuspended Protein A + G Agarose at 4°C for 2 h on a rotator. Centrifugation at 300×*g* for 5 min, and the supernatant was collected and incubated with 20 μg anti‐RSPO3 antibody at 4°C for 12 h. The mixtures were then incubated with Protein A + G Agarose at 4°C for 2 h on a rotator. The complexes were washed twice with IP washing buffer. The washed beads were incubated with 1X SDS‐polyacrylamide gel electrophoresis loading buffer and boiled at 100°C for 10 min to prepare the proteins sample that were subsequently 21transferred onto the gel using SDS‐PAGE.

### 2.9 In‐gel digestion

The gel strips were chopped and destained in 50% acetonitrile containing NH_4_HCO_3_, dehydrated with 100% acetonitrile, rehydrated in dithiothreitol. Gel pieces were again dehydrated in 100% acetonitrile, and rehydrated with iodoacetamide. Gel pieces were rehydrated with trypsin resuspended in NH_4_HCO_3_. Liquid was removed and gel pieces were digested with trypsin. The peptide solution was frozen and spinned dry for liquid chromatography coupled with tandem mass spectrometry (LC–MS/MS).

### 2.10 LC–MS/MS analysis

Peptides were dissolved informic acid (solvent A) and loaded onto a reversed‐phase analytical column. The gradient comprised of an increase of 6%–23% in solvent B (0.1% formic acid in 98% acetonitrile) over 16 min, 23%–35% in 8 min, climbing to 80% in 3 min then staying at 80%, at a constant flow rate of 400 nL/min on an EASY‐nLC 1000 UPLC system (Thermo Fisher Scientific, Inc., USA). Following separation using the UPLC system, peptides were subjected to MS/MS in Q ExactiveTM Plus (Thermo Fisher Scientific, Inc., USA). The full m/z scan range was 350–1800, and peptides and the fragments were detected in the Orbitrap at a resolution of 17,500. One MS scan was followed by 20 MS/MS scans with 15.0 sec dynamic exclusion. Automatic gain control was set at 5E4.

### 2.11 Data processing

The MS/MS data was processed using Proteome Discoverer 1.3. Trypsin/P was specified as a cleavage enzyme allowing up to two missing cleavages. Mass error was set to 10 ppm for precursor ions and 0.02 Da for fragment ions. Peptide ion score was set to >20, and peptide confidence was set at high.

### 2.12 Immunofluorescence staining of β‐catenin

Both MKN45 and AGS cells were labeled with an antibody against β‐catenin (cat. no. 8480S, Cell Signaling Technology, Inc., USA). The bound antibodies were detected by an FITC‐labeled (red) secondary antibody (cat. no. A0562, Beyotime Institute of Biotechnology, China). The nuclei were stained with 4′,6‐diamidino‐2‐phenylindole DAPI (cat. no.C1005, Beyotime Institute of Biotechnology, China) and the cells were observed under a fluorescence microscope.

### 2.13 Animal studies

All of the experiments were carried out according to the animal protocol approved by the Institutional and Local Committee on the Care and Use of Animals of Shanghai University of Traditional Chines Medicine (Ethical approval number, PZSHUTCM210122003). Twenty age‐matched nude mice (6–8 weeks old, obtained from Shanghai SLAC laboratory animal Co. Ltd) were used, 10 in each group. A total of 3 × 10^6^ sh‐RSPO3 (The level of RSOP3 was downregulated approximately 83.12% as compared to scramble MKN45 cells) or sh‐scramble MKN45 cells in 100 μL PBS were intraperitoneally injected respectively. To ensure human endpoints to determine whether animals should be euthanized, the mental state, food intake, activity and weight of mice were monitored once a week. After 4 weeks, mice were euthanized by CO_2_ asphyxiation (CO_2_ was introduced at a rate of 50% of the chamber volume per min), and followed by cervical dislocation. Following mouse euthanasia. Abdominal metastatic tumors were counting and measuring the size of each tumor, tissue samples were collected for pathological analysis.

### 2.14 Immunohistochemistry (IHC)

Animal GC tissues sections were detected. Following deparaffinization, sections were blocked for endogenous peroxidase and transferred to an antigen retrieval solution. Sections were then incubated with antibodies against RSPO3, CD44, SOX‐2, Nanog, and OCT‐4 (dilution, 1:100). Following incubation with the secondary antibody (cat. no. A0208, Beyotime Institute of Biotechnology, China), DAB was used to illuminate the positive staining signals, and counterstained with hematoxylin. ImageJ (National Institutes of Health, Bethesda, USA) analyzed the positive staining signals.

### 2.15 Statistical analysis

GraphPad Prism 8 (GraphPad Software, Inc.) analyzed the statistical data. One‐way ANOVA analyzed any significant differences. *p* < 0.05 indicate a statistically significant difference.

## 3 RESULTS

### 3.1 GC cells cocultured with *H. pylori* extracts generates cells with stem cell properties

*H. pylori* extracts were cocultured with the AGS and MKN45 GC cell lines to determine whether it influenced CSC properties. Specific markers could be detected to identify CSC properties, such as CD44,^27^ SOX‐2,^28^ Nanog,^29^ and OCT‐4.^30^ In vitro formation of three‐dimensional spheroids highlights the capacity of cells for self‐renewal, which is ideal for the identification of CSC properties.

**FIGURE 1:**
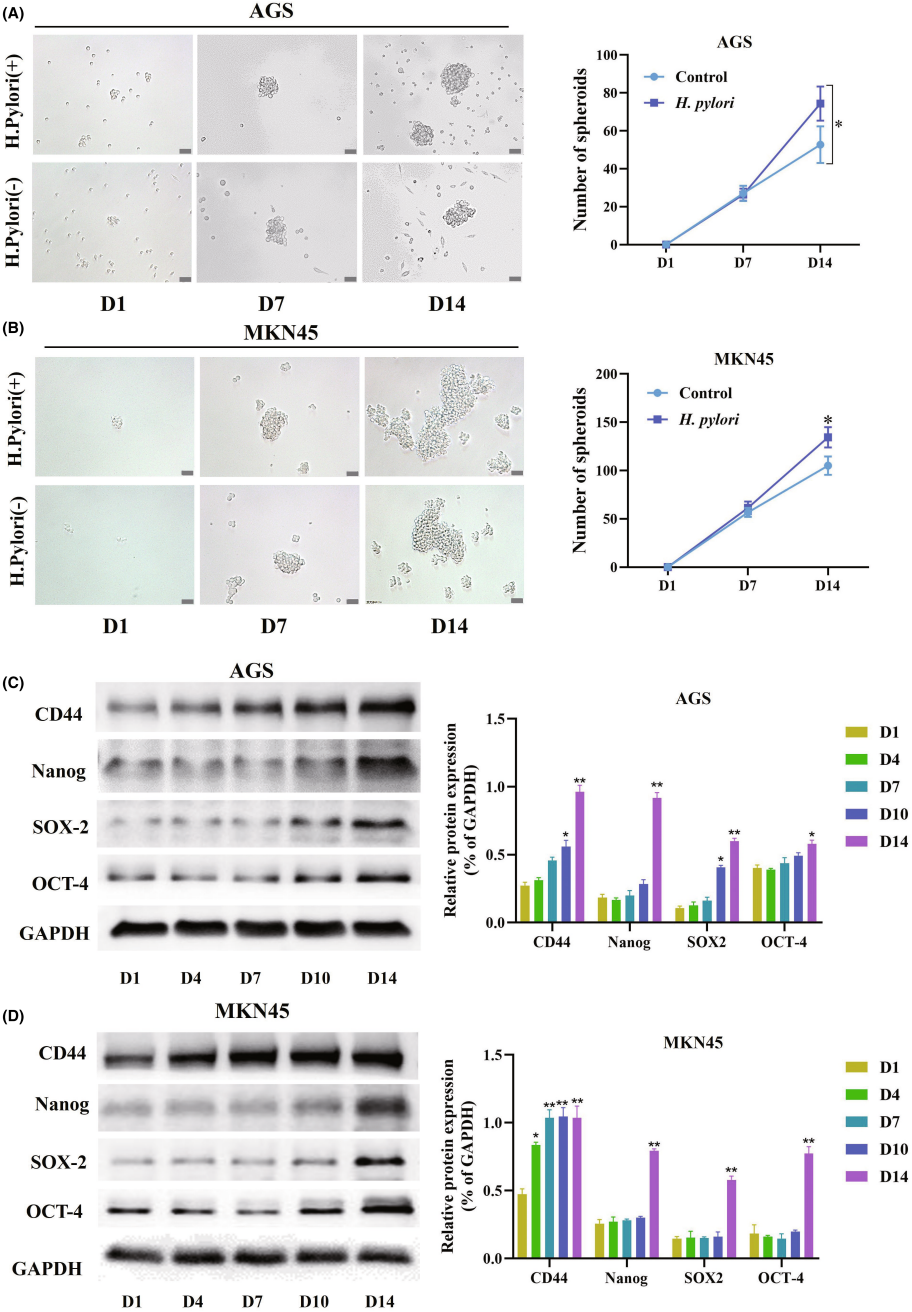
GC cells cocultured with *H. pylori* extracts stimulates stemness. (A, B) AGS and MKN45 cells were seeded under non‐adherent culture conditions for spheroid formation for 2 weeks. Scale bar = 50 μm. (C, D) The protein levels of CD44, Nanog, SOX‐2, and OCT‐4 were analyzed by western blotting in AGS and MKN45 cells. All experiments were repeated three times, *p < 0.05 and **p < 0.01 versus control. GC, gastric cancer; Nanog, Nanog homeobox; OCT‐4, organic cation/carnitine transporter‐4; SOX‐2, SRY‐box transcription factor 2.

The CSC properties of AGS and MKN45 cells were first investigated in vitro for spheroid formation. Both AGS and MKN45 cells could spontaneously form tumor spheres after 1 day of culture under non‐adherent conditions in special media. One week after cocultured with *H. pylori*, no significant increase was observed in spheroid formation or enlargement, but on the 10th day, spheroid formation increased and spheres were enlarged significantly, as compared with uninfected control GC cells (Figure 1A,B). In addition, markers of CSC properties (CD44, SOX‐2, Nanog, and OCT‐4) were also identified. In accordance with the spheroid formation experiments, CD44 expression started to increase on the 4th day, with the expression of all markers was significantly elevated on the 10th day (Figure 1C,D). But in *H.Pylori* (−) spheroids, most of these markers barely exhibited change trend (data not shown). Therefore, the CSC properties of GC cells could be increased after coculturing with *H. pylori*.

### 3.2 RSPO3 is elevated by H. pylori and promotes CSC properties

**FIGURE 2:**
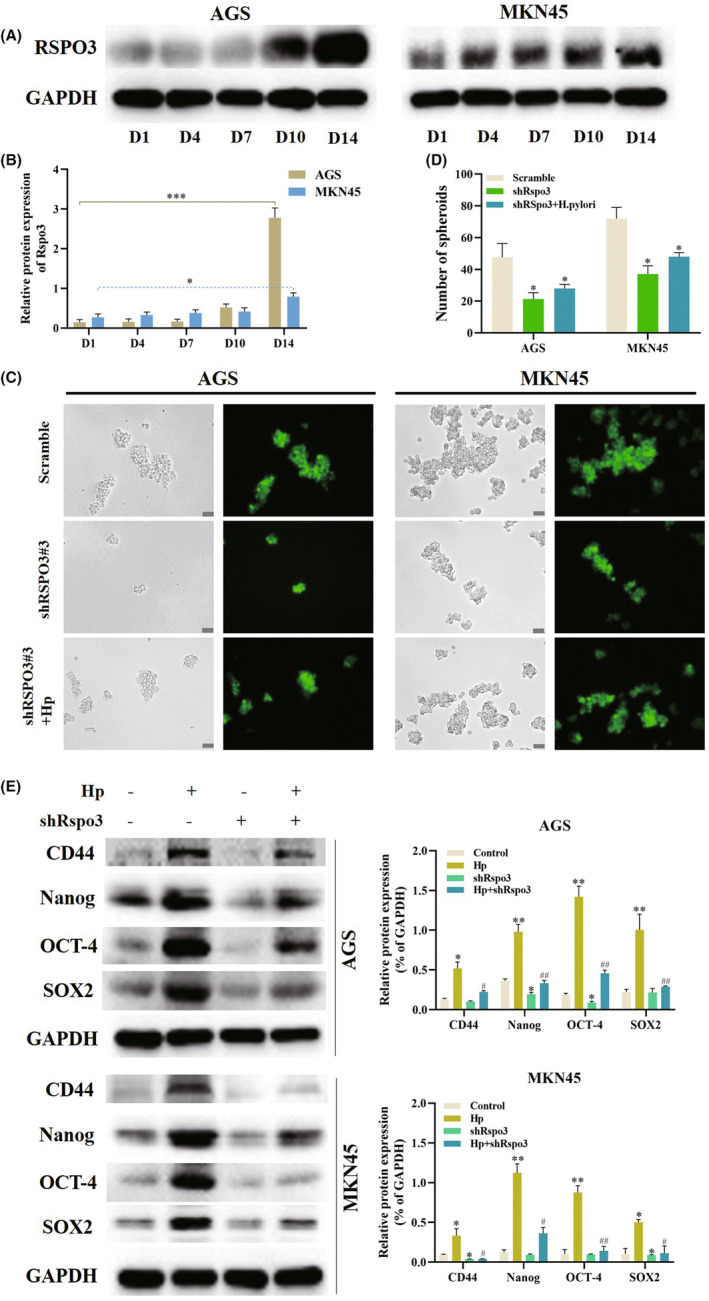
RSPO3 is elevated by *H. pylori* and promotes GC stem cell properties. (A, B) The protein levels of RSPO3 in AGS and MKN45 cells were analyzed by western blotting. (C, D) sh‐RSPO3#3 AGS and MKN45 cells were seeded under non‐adherent culture conditions for spheroid formation for 2 weeks. Scale bar = 50 μm. (E) The protein levels of CD44, Nanog, SOX‐2, and OCT‐4 in AGS and MKN45 cells were analyzed by western blotting. All experiments were repeated three times, *p < 0.05, **p < 0.01, ^#^ *p* < 0.05 and ^##^ *p* < 0.01 versus Hp. GC, gastric cancer; Nanog, Nanog homeobox; OCT‐4, organic cation/carnitine transporter‐4; RSPO3, R‐spondin 3; SOX‐2, SRY‐box transcription factor 2.

The protein expression of RSPO3 was detected in GC cells on the 1st, 4th 7th, 10th, and 14th day after after coculturing with *H. pylori*. Of note, RSPO3 expression started to increase on the 10th day, and was significantly increased on the 14th day in both GC cell lines (Figure 2A,B), which was consistent with the change trend of CSC markers. To determine whether RSPO3 is indispensable in CSC properties, stable sublines of GC cells that reduced the expression of RSPO3 (sh‐RSPO3#1, sh‐RSPO3#2, and sh‐RSPO3#3) were developed. The tight regulation of the green fluorescent protein (GFP) marker was achieved through the incubation with puromycin, and the levels of RSPO3 were readily suppressed by sh‐RSPO3#3 (Figure S1).

Next, whether RSPO3 influences CSC properties, as well as the relationship between RSPO3 and *H. pylori* were explored. Compared with sh‐scramble cells, sh‐RSPO3#3 cells grown on ultra‐low attachment culture plates formed less and much smaller spheroids after 2 weeks, but when cocultured with *H. pylori*, sh‐RSPO3#3 cells partly regained the ability to grow more and bigger spheroids (Figure 2C,D). In addition, markers of CSC properties, CD44, SOX‐2, Nanog, and OCT‐4 were also reduced in sh‐RSPO3#3 cells. Of note, sh‐RSPO3#3 significantly decreased the *H. pylori*‐induced increase in the expression of CSC markers (Figure 2E).

### 3.3 RSPO3 facilitates β‐catenin's translocation into the nucleus without Wnts

**FIGURE 3:**
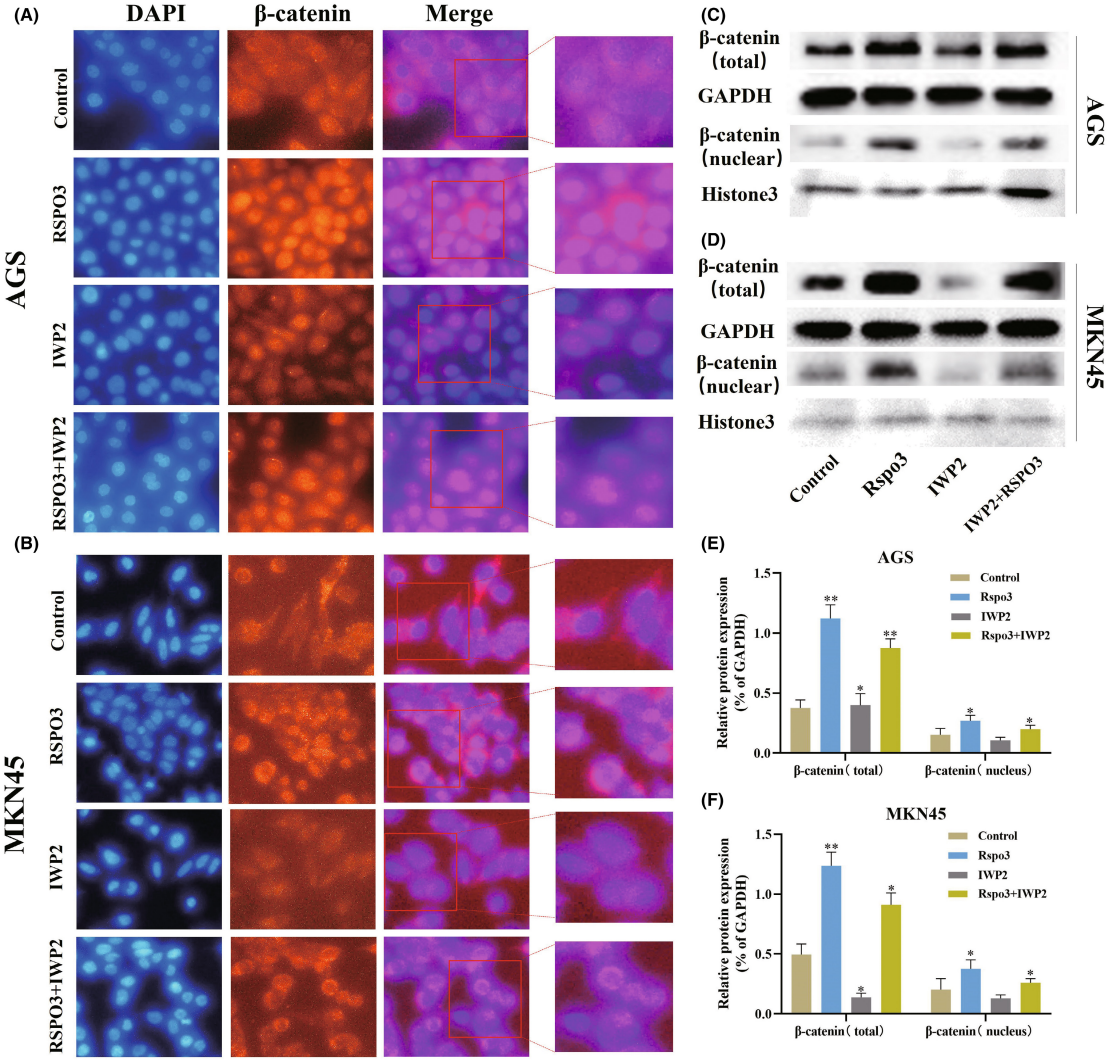
RSPO3 facilitates the β‐catenin translocation into the nucleus without Wnts. (A, B) Immunofluorescence image of β‐catenin in AGS and MKN45 cells. Scale bar = 50 μm. (C–F) Proteins levels of β‐catenin in AGS and MKN45 cells were analyzed by western blotting. All experiments were repeated three times, *p < 0.05 and **p < 0.01 versus scramble. RSPO3, R‐spondin 3.

The Wnt signaling pathway contributes to the regulation of CSC properties.^31^ Activation of β‐catenin is instrumental in GC spheroid formation and tumor‐initiating capacity,^32^ and previous reports have demonstrated that RSPO3 potentiates the Wnt/β‐catenin signaling pathway.^33^, ^34^ Inhibitors of Wnt production‐2 (IWP‐2) targets the membrane‐bound O‐acyltransferase porcupine, thus inhibiting the Wnt/β‐catenin signaling pathway,^35^ so we used IWP2 (5 μM) to inhibit Wnt signaling pathway. Immunofluorescence showed that recombinant RSPO3 protein increased the expression of β‐catenin and facilitated its translocation into the nucleus, while the expression of β‐catenin was decreased when cultured with IWP2. Of note, the expression of β‐catenin remains increased by recombinant RSPO3 protein when cocultured with IWP2 (Figure 3A,B). Western Blotting also support the above results (Figure 3C–F), which suggested that RSPO3 may regulate β‐catenin through a Wnt‐independent pathway.

### 3.4 RSPO3 interacts with GNG7 to activate the phosphatidylinositol 3 kinase/protein kinase B (PI3K/Akt)/GSK‐3β/β‐catenin pathway and promote GC stemness

As Wnt is not the only response ligand of RSPO3 in GC cells. CoIP and LC–MS/MS were then performed to find the interacting proteins with RSPO3. According to the CoIP and LC–MS/MS results, it was found that G protein gamma 7 (GNG7) interacted with RSPO3 (Table S1). GNG7 is one of the G protein βγ subunits that directly activate the PI3K/Akt pathway.^36^, ^37^ Phosphorylation of GSK‐3β activates PI3K/Akt signaling and lead to β‐catenin stabilization.^38^, ^39^ Therefore, we hypothesized that RSPO3 may activate the PI3K/Akt pathway by binding to GNG7, thus promoting the phosphorylation of GSK‐3β and activation of β‐catenin.

Next the interaction between the RSPO3 and GNG7 proteins was assessed using IP and western blotting, and the results confirmed that RSPO3 directly interacted with GNG7 (Figure S2). Besides, the immunofluorescent staining exhibited the co‐localization of GNG7 and RSPO3 (Figure S3), GNG7 was mainly expressed on the cell membrane (fluorescence in red), while Rspo3 was distributed in the cytoplasm and cell membrane (fluorescence in green), and some regions of these two proteins showed overlap. So it could be speculated that Rspo3 and GNG7 at least interact intracellularly. We then detect the protein expression of GNG7 in GC cells on the 1st, 4th 7th, 10th, and 14th day after coculturing with *H. pylori* extracts. Interestingly, GNG7 expression increase on the 14th day in both GC cell lines (Figure S4), which was started to rise after the change trend of RSPO3.

**FIGURE 4:**
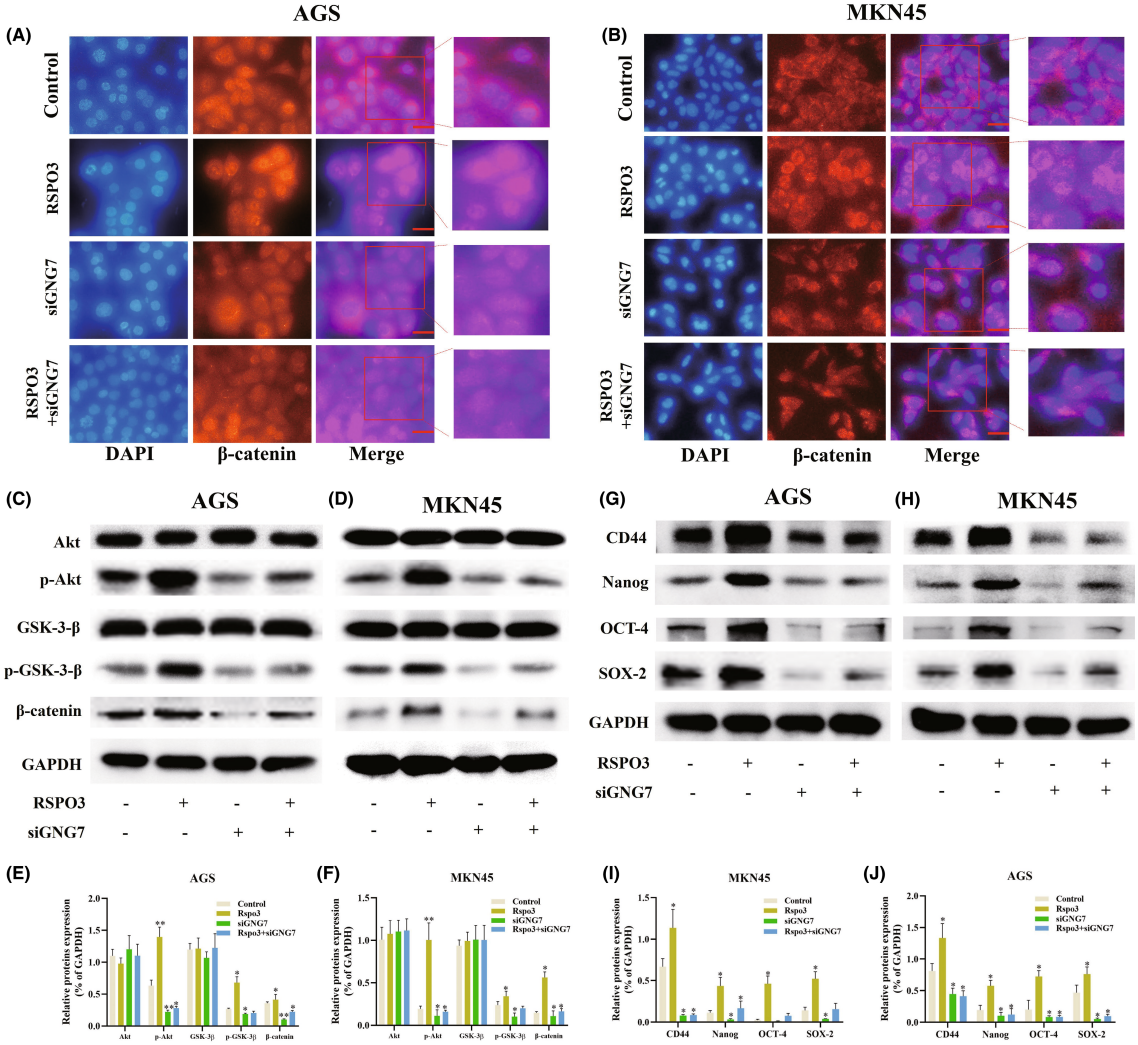
RSPO3 interacts with GNG7 to activate the PI3K/Akt/GSK‐3β/β‐catenin pathway and promote GC stemness. (A, B) Immunofluorescence image of β‐catenin in AGS and MKN45 cells. Scale bar = 50 μm. (C–F) Protein levels of Akt, p‐Akt, GSK‐3β, and p‐GSK‐3β in AGS and MKN45 cells, and protein levels of β‐catenin in cells were analyzed by western blotting. *p < 0.05 and **p < 0.01 versus Control. (G–J) Protein levels of CD44, Nanog, OCT‐4 and SOX‐2 in AGS and MKN45 cells were analyzed by western blotting. All experiments were repeated three times, *p < 0.05 and **p < 0.01 versus scramble. GC, gastric cancer; GNG7, G protein subunit gamma 7; GSK‐3β, glycogen synthase kinase‐3β; Nanog, Nanog homeobox; OCT‐4, organic cation/carnitine transporter‐4; RSPO3, R‐spondin 3.

To determine whether GNG7 is an effector of RSPO3/β‐catenin in *H. pylori* extracts, the si‐RNA‐mediated knockdown of GNG7, si‐GNG7#1, si‐GNG7#2, and si‐ GNG7#3 was performed. The level of GNG7 was suppressed by si‐GNG7#3 (Figure S5). Of note, recombinant RSPO3 protein increased the phosphorylation level of Akt and GSK‐3β in GC cells. The expression of β‐catenin was also elevated, but this regulatory effect of RSPO3 could be blocked by si‐GNG7 (Figure 4A–F), suggesting that RSPO3 activates β‐catenin via GNG7. Besides, AKT and GSK3β phosphorylation were increased by *H. pylori* extracts, and were decreased by shRSPO3. Of note, shRSPO3 could diminish the promoting effect of *H. pylori* extracts to the AKT and GSK3β phosphorylation (Figure S6).

To determine whether the RSPO3‐induced promotion of GC stemness is mediated by GNG7, GNG7 was suppressed by si‐GNG7#3 and cocultured with recombinant RSPO3 protein in GC cells. Consistent with the previous data, stemness markers (CD44, Nanog, Oct‐4, and SOX‐2) were significantly increased by recombinant RSPO3 protein, but were decreased by GNG7 suppression (Figure 4G–J). Of note, GNG7 suppression could diminish the promoting effect of RSPO3 to stemness, which was consistent with the change in β‐catenin. Therefore, RSPO3 enhanced the stemness of *H. pylori* extracts‐infected GC cells through the GNG7/β‐catenin signaling pathway.

### 3.5 Inhibition of RSPO3 suppresses GC tumor peritoneal seeding in vivo

Next, it was determined whether RSPO3 influences GC tumor metastasis in vivo, Peritoneal metastasis is the most common cause of mortality for GC,^40^ and GC cell spheroids are more likely to successfully achieved colonization to the abdomen.^41^ Therefore, whether RSPO3 inhibition would inhibit GC tumor peritoneal seeding in vivo was explored.

**FIGURE 5:**
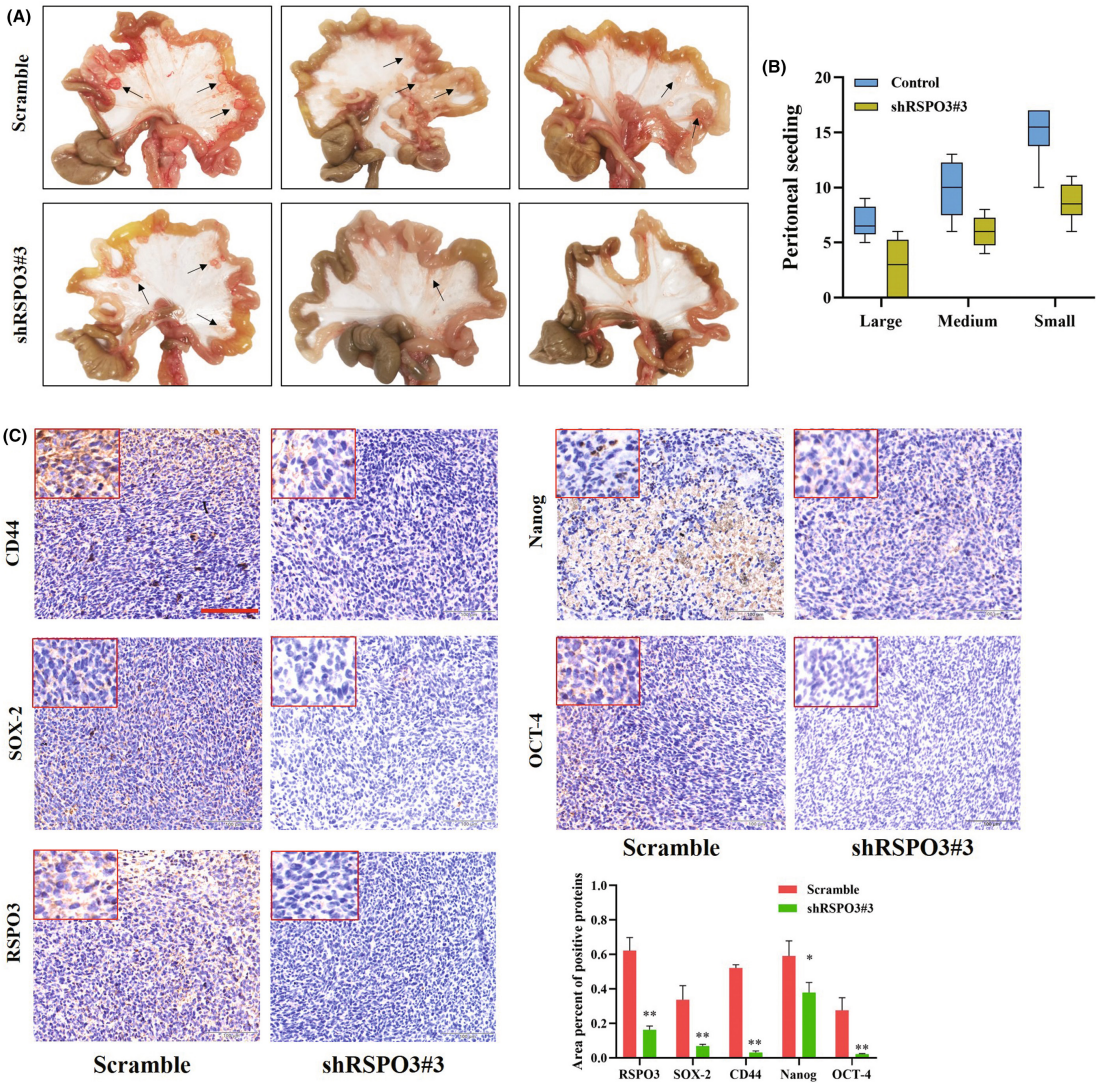
RSPO3 suppression inhibits MKN45 tumor peritoneal seeding in vivo. (A) In an MKN45 tumor peritoneal seeding model, sh‐RSPO3#3 exhibited a significant inhibitory effect on peritoneal tumor metastasis. (B) Measurement of peritoneal tumor metastasis at the conclusion of the study. (C) Immunohistochemical staining of tumor specimens for RSPO3, SOX‐2, CD44, Nanog, and OCT‐4. A positive protein area was detected by DAB chromogen (brown) (magnification, ×200). Inserts showing a higher magnification (magnification, ×400). Scale bar = 100 μm. *p < 0.05 and **p < 0.01 versus scramble. Nanog, Nanog homeobox; OCT‐4, organic cation/carnitine transporter‐4; RSPO3, R‐spondin 3; SOX‐2, SRY‐box transcription factor 2.

Intraperitoneal implantation of MKN45 cells resulted in a widespread of seeded metastases throughout the peritoneal cavity. In the ^RSPO3‐^GC group, the number of bulk tumor seedings was significantly lower than that in the control group (Figure 5A). The number of small (1‐mm), large (>1‐mm, <3‐mm), and bulk (>3‐mm) peritoneal tumor seedings was quantified, and is shown in Figure 5B. IHC detection also proved that RSPO3 expression was significantly weaker in ^RSPO3‐^metastatic tumors as compared to control group. CD44 showed a significant decrease in ^RSPO3‐^metastatic tumors (*p* < 0.001), and CD44, OCT‐4, and SOX‐2 staining was weaker in ^RSPO3‐^metastatic tumors (Figure 5C). In combination, these studies revealed the role of RSPO3 in the metastasis of peritoneal tumors.

## 4 DISCUSSION

*H. pylori* can colonize deep inside gastric glands, followed by triggering RSPO3 signaling.^42^ However, it is unclear whether RSPO3 promote the development of GC. The present study revealed that the aberrant RSPO3 expression, which can be triggered by *H. pylori* extracts, could promote stemness that leads to GC development. In addition, exogenous recombinant human RSPO3 also promotes GC cell stemness. It was also showed that RSPO3 knockdown significantly reduced the *H. pylori‐*induced CSC‐like properties. These findings suggested a possible mechanism underlying the relationship between RSPO3 and GC tumor evolution.

The hyperactivation of the Wnt signaling pathway has been found to induce the nuclear translocation of β‐catenin, and enhance the self‐renewal of CSCs. Although previous studies have demonstrated that RSPO3 potentiates the Wnt/β‐catenin signaling pathway,^18^, ^19^, ^33^, ^43^, ^44^, ^45^, ^46^, ^47^, ^48^, ^49^, ^50^, ^51^, ^52^, ^53^, ^54^, ^55^, ^56^, ^57^, ^58^, ^59^, ^60^, ^61^ but a different study reported it could potentiate β‐catenin without LGRs.^62^ Coincidentally, we found that the inhibitor of Wnt(IWP‐2) can't diminish the RSPO3‐induced activation of β‐catenin. The above results suggested that RSPO3 may regulate β‐catenin through a Wnt‐independent pathway.

To further explore the mechanism through which RSPO3 regulates CSC‐like properties, CoIP, and LC–MS/MS were performed to identify the proteins interacting with RSPO3. Some proteins were identified, and we focused on GNG7, as it has been reported to directly activate PI3K/Akt pathway.^63^ The inhibition of an important PI3K/Akt signaling substrate, GSK‐3β, through its phosphorylation by activating PI3K/Akt signaling can lead to β‐catenin stabilization and nuclear translocation for next gene transcription.^38^, ^39^ Therefore, we hypothesized that RSPO3 may activate the PI3K/Akt pathway by binding to GNG7 on the cell membrane, thus promote the phosphorylation of GSK‐3β and the activation of β‐catenin.

Next, the interaction between the RSPO3 and GNG7 proteins was assessed by IP and western blotting, and the results further confirmed that RSPO3 directly interacted with GNG7. To determine whether GNG7 is an effector of RSPO3/β‐catenin in *H. pylori* extracts, si‐RNA‐mediated GNG7 knockdown was applied. Of note, recombinant RSPO3 protein increased the phosphorylation level of Akt and GSK‐3β in GC cells, and the expression of β‐catenin was increased, but this regulatory effect of RSPO3 could be partly blocked by si‐GNG7, suggesting that RSPO3 activates β‐catenin via GNG7.

To determine whether the RSPO3‐induced promotion of GC stemness is mediated by GNG7, GNG7 was suppressed and cocultured with recombinant RSPO3 protein in GC cells. Recombinant RSPO3 protein could significantly increase the expression of all stemness markers (CD44, Nanog, OCT‐4, and SOX‐2). Of note, GNG7 suppression could diminish the promoting effect of RSPO3 on stemness, which was consistent with the change of β‐catenin. In addition, RSPO3 suppression inhibits MKN45 tumor peritoneal seeding and ascites in vivo, which accompanied by improving the animal health, behavior and weight of mice. Therefore, RSPO3 enhanced *H. pylori* extracts infected GC cells stemness through the GNG7/β‐catenin signaling pathway.

In conclusion, our research indicated that *H. pylori* promoted CSC‐like properties. RSPO3, which can be ignited by *H. pylori* extracts, promotes the CSC‐like phenotype. RSPO3 knockdown significantly reduced the *H. pylori‐*induced CSC‐like properties. GNG7 is one of the transmembrane proteins interacting with RSPO3. Recombinant RSPO3 protein increased the phosphorylation level of Akt, GSK‐3β, and the expression of β‐catenin in GC cells, but this regulatory effect of RSPO3 could be blocked by GNG7 knockdown. Of note, GNG7 suppression could diminish the promoting effect of RSPO3 on stemness. In addition, RSPO3 suppression inhibits MKN45 tumor peritoneal seeding and ascites in vivo. IHC staining also showed that RSPO3, CD44, OCT‐4, and Nanog were increased in *H. pylori* GC tissues. Collectively, RSPO3 enhanced the stemness of *H. pylori* extracts‐infected GC cells through the GNG7/β‐catenin signaling pathway. Our research reveal the new sight of how *H. pylori* promote GC progression.

## AUTHOR CONTRIBUTIONS

**Xiwu Rao:** Conceptualization (equal); data curation (equal); formal analysis (equal). **Hao Hu:** Writing – review and editing (equal). **Zhipeng Zhang:** Methodology (equal); visualization (equal). **Yunzhou Pu:** Investigation (equal); writing – original draft (equal). **Gang Han:** Project administration (equal). **Hangjun Gong:** Supervision (equal); validation (equal). **Qing Ji:** Conceptualization (equal); writing – review and editing (equal). **Ningning LIU:** Project administration (equal).

## FUNDING INFORMATION

This work was supported by Natural Science Foundation of Shanghai (19ZR1458500) and China Postdoctoral Science foundation (no. 2023M740860).

## CONFLICT OF INTEREST STATEMENT

The authors declare that they have no competing interests.

## ETHICS STATEMENT

The animal studies were reviewed and approved by the Ethics Committee for Animal Studies at Shanghai University of Traditional Chines Medicine (Ethical approval number: PZSHUTCM210122003).

## CONSENT FOR PUBLICATION

All authors agreed on the manuscript.

## Supporting information


Figure S1.
Figure S2.
Figure S3.
Figure S4.
Figure S5.
Figure S6.

Table S1.

## Data Availability

The datasets used and/or analyzed during the current study are available from the corresponding author on reasonable request.
