# Supplementary material for: RSPO3 induced by Helicobacter pylori extracts promotes gastric cancer stem cell properties through the GNG7/β‐catenin signaling pathway
Source: Cancer Med. 2024 Apr 5;13(7):e7092. doi: 10.1002/cam4.7092 (PMC10997846; doi:10.1002/cam4.7092)
Supplement: Supplementary file 1 — Figure S1. Figure S2. Figure S3. Figure S4. Figure S5. Figure S6. [file CAM4-13-e7092-s001.pdf]

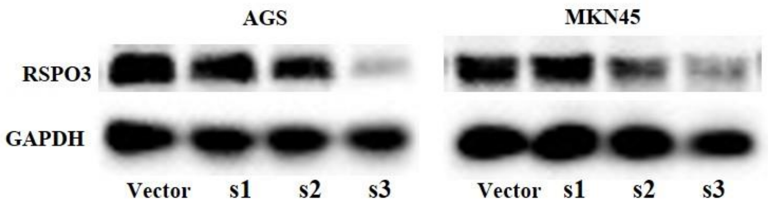

Fig.S1 Levels of RSPO3 were suppressed by sh-RSPO3#3 in GC cells

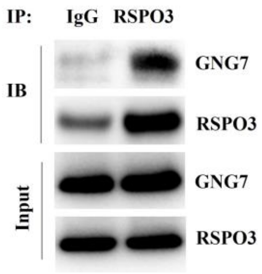

Fig.S2 RSPO3 directly interacted with GNG7 in MKN45 cell

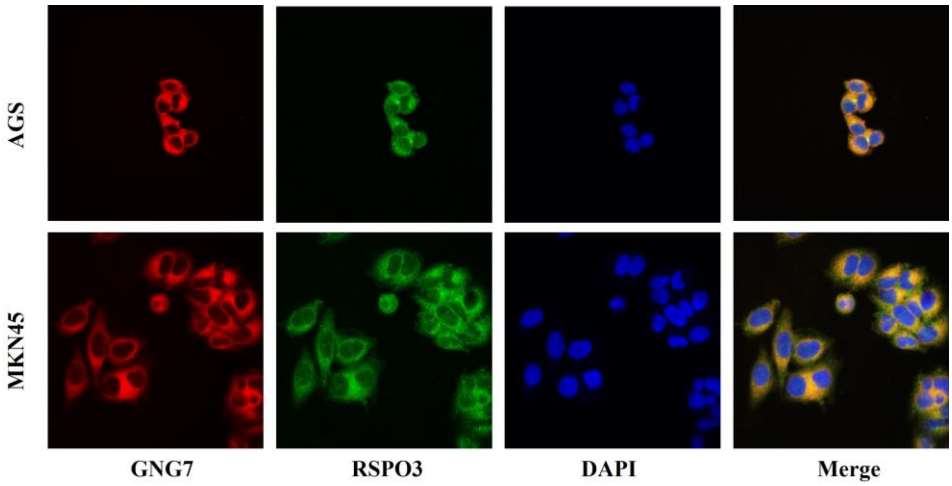

Fig.S3 Co-localization of GNG7 and RSPO3 by immunofluorescent staining in GC cells

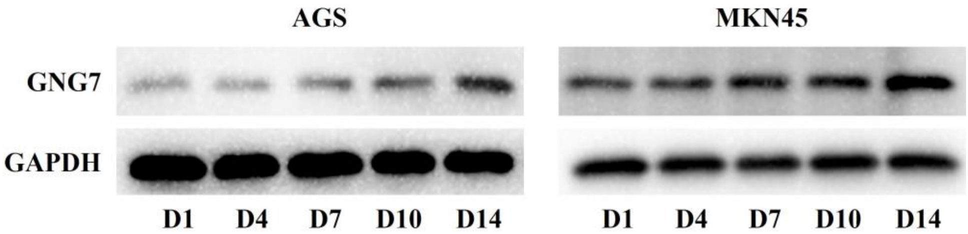

Fig.S4 Levels of GNG7 is elevated by H. pylori extracts in GC cells

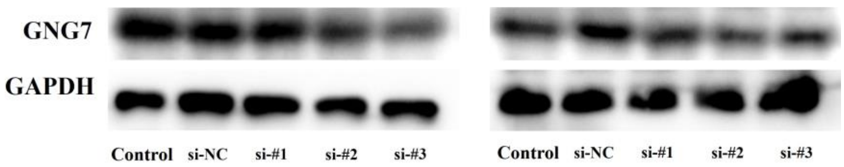

Fig.S5 Levels of GNG7 were suppressed by si-GNG7#3 in GC cells

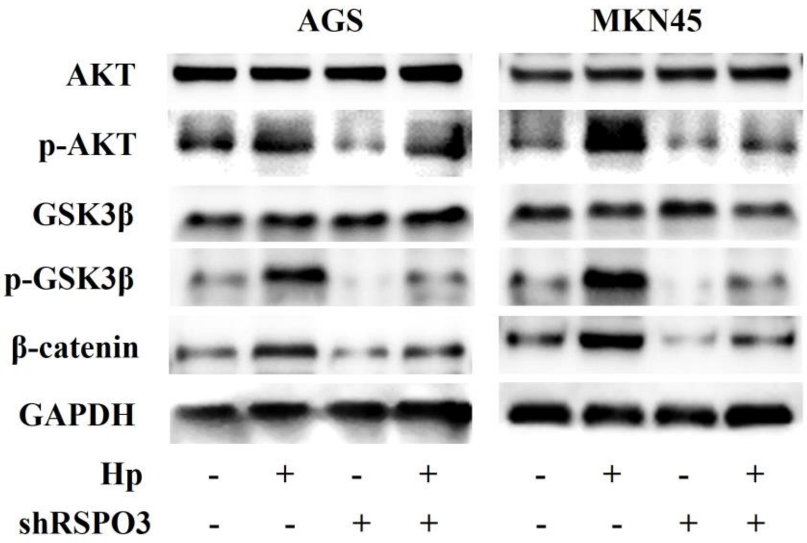

Fig.S6 Protein levels of Akt, p-Akt, GSK-3β and p-GSK-3β, β-catenin in GC cells were analyzed by western blotting
