# Supplementary material for: RSPO3 induced by Helicobacter pylori extracts promotes gastric cancer stem cell properties through the GNG7/β‐catenin signaling pathway
Source: Cancer Med. 2024 Apr 5;13(7):e7092. doi: 10.1002/cam4.7092 (PMC10997846; doi:10.1002/cam4.7092)
Supplement: Supplementary file 2 — Table S1. [file CAM4-13-e7092-s002.docx]

| **CoIP and LC-MS/MS analysis identified proteins interacting with RSPO3** | | | | | | | | |
| --- | --- | --- | --- | --- | --- | --- | --- | --- |
| **Accession** | **Protein names** | **Gene names** | **MW [kDa]** | **Protein score** | **Sequence coverage (%)** | **Unique Peptides** | **Peptides** | **PSMs** |
| P60709 | Actin, cytoplasmic 1 | ACTB | 41.709 | 93.459 | 6.93 | 2 | 2 | 2 |
| P02768 | Serum albumin | ALB | 69.321 | 85.888 | 3.61 | 3 | 3 | 3 |
| Q9NZT1 | Calmodulin-like protein 5 | CALML5 | 15.882 | 57.3 | 9.59 | 1 | 1 | 1 |
| Q15834 | Coiled-coil domain-containing protein 85B | CCDC85B | 22.077 | 45.88 | 3.47 | 1 | 1 | 1 |
| P81605 | Dermcidin | DCD | 11.28 | 53.42 | 10.00 | 1 | 1 | 1 |
| P15924 | Desmoplakin | DSP | 331.57 | 90.25 | 0.94 | 3 | 3 | 3 |
| Q5D862 | Filaggrin-2 | FLG2 | 247.93 | 89.09 | 0.50 | 1 | 1 | 1 |
| O60262 | Guanine nucleotide-binding protein G(I)/G(S)/G(O) subunit gamma-7 | GNG7 | 7.52 | 54.18 | 10.29 | 1 | 1 | 1 |
| Q86YZ3 | Hornerin | HRNR | 282.23 | 251.32 | 13.75 | 8 | 8 | 8 |
| P38646 | Stress-70 protein, mitochondrial | HSPA9 | 73.63 | 49.02 | 1.33 | 1 | 1 | 1 |
| P01877 | Immunoglobulin heavy constant alpha 2 | IGHA2 | 36.57 | 66.95 | 2.94 | 1 | 1 | 1 |
| P04264 | Keratin, type II cytoskeletal 1 | KRT1 | 66.00 | 1007.22 | 41.15 | 18 | 21 | 32 |
| P13645 | Keratin, type I cytoskeletal 10 | KRT10 | 58.79 | 736.05 | 29.79 | 17 | 19 | 24 |
| P02533 | Keratin, type I cytoskeletal 14 | KRT14 | 51.53 | 356.73 | 24.79 | 5 | 10 | 12 |
| P08779 | Keratin, type I cytoskeletal 16 | KRT16 | 51.24 | 298.64 | 20.30 | 5 | 9 | 11 |
| Q04695 | Keratin, type I cytoskeletal 17 | KRT17 | 48.076 | 131.450 | 9.49 | 1 | 4 | 4 |
| P35908 | Keratin, type II cytoskeletal 2 epidermal | KRT2 | 65.393 | 906.546 | 51.49 | 22 | 25 | 28 |
| P13647 | Keratin, type II cytoskeletal 5 | KRT5 | 62.339 | 202.130 | 14.41 | 6 | 8 | 8 |
| P02538 | Keratin, type II cytoskeletal 6A | KRT6A | 60.008 | 178.688 | 14.54 | 1 | 8 | 8 |
| P48668 | Keratin, type II cytoskeletal 6C | KRT6C | 59.988 | 178.841 | 14.54 | 1 | 8 | 8 |
| P35527 | Keratin, type I cytoskeletal 9 | KRT9 | 62.026 | 1173.371 | 51.2 | 23 | 23 | 30 |
| P31025 | Lipocalin-1 | LCN1 | 19.237 | 94.575 | 12.5 | 2 | 2 | 2 |
| Q6UWP8 | Suprabasin | SBSN | 60.504 | 56.05 | 9.15 | 1 | 1 | 1 |
| P29508 | Serpin B3 | SERPINB3 | 44.536 | 52.92 | 1.79 | 1 | 1 | 1 |
| Q08188 | Protein-glutamine gamma-glutamyltransferase E | TGM3 | 76.583 | 50.83 | 1.3 | 1 | 1 | 1 |
